# Characterization of a new low‐dose and low‐energy Gafchromic film LD‐V1

**DOI:** 10.1002/acm2.14531

**Published:** 2024-09-11

**Authors:** Oliva Masella, Kevin J. Murphy, Magdalena Bazalova‐Carter

**Affiliations:** ^1^ Physics and Astronomy Department University of Victoria Victoria British Columbia Canada

**Keywords:** dosimetry, film, low energy x‐rays

## Abstract

**Purpose:**

To characterize the dose‐response, energy dependence, postexposure changes, orientation dependence, and spatial capabilities of LD‐V1, a new low‐dose Gafchromic film for low‐energy x‐ray dosimetry.

**Methods:**

A single sheet of LD‐V1 Gafchromic film was cut into 15 × 20 mm^2^ rectangles with a notch to track orientation. Eight different doses between 5 and 320 mGy were delivered by an MXR‐160/22 x‐ray tube using x‐ray beams of 90, 100, and 120 kVp filtered with 3 mm of Al and 2 mm of Ti. The 120 kVp films were scanned at 1, 1.5, 2, 3, 12, 24, 48, 72, and 168 h postexposure in portrait orientation and additionally scanned in landscape orientation at 24 h. The 90 and 100 kVp films were scanned at 24 h postexposure in portrait orientation. Lastly, a 20 × 200 mm^2^ strip of film was irradiated using a thin‐slit imaging collimator and scanned 24 h postexposure to test the film performance in an x‐ray imaging application.

**Results:**

Of the three color channels, the red channel was found to produce a dose‐response curve with a large range of net optical density (netOD) values across the considered dose range. A prominent energy dependence was discovered, resulting in dose discrepancies on the scale of 17 mGy between 90 and 120 kVp for a dose of 80 mGy. The measured postexposure changes suggest that the calibration irradiation‐to‐scan time should be longer than 12 h with a ± 4 h scanning time window for dose errors of <0.5%. An average dose difference of 3.4% was found between the two scanning orientations. Lastly, noise of 4% was measured in the thin slit collimator film for a dose of 30 mGy.

**Conclusions:**

We have characterized the LD‐V1 film for low‐energy, low‐dose x‐ray dosimetry. Energy, scan‐time, and orientation dependencies should be considered when using this film.

## 1 INTRODUCTION

Radiochromic film is a valuable tool for radiotherapy dosimetry. Thanks to their similar radiological properties to water and small *<*0.5 mm thickness, the presence of films has a minimal impact on dose measurements.^1^ In the clinic, films offer reliable quality assurance testing, particularly in cases where high spatial precision and dosimetric accuracy are needed as with linac commissioning,^1^ diagnostic equipment testing,^2^, ^3^ and the validation of dose distributions delivered using various clinical techniques such as intensity‐modulated radiation therapy (IMRT), MRI‐guided radiation therapy (MR‐IGRT), and stereotactic radiation therapy (SRT).^4^, ^5^, ^6^, ^7^ Meanwhile, films are often employed in research for the verification of dose delivery for novel equipment or techniques, which makes films an invaluable tool for validation and proof of principle experiments.^8^, ^9^, ^10^, ^11^

The main producer of Gafchromic films Ashland (Bridgewater, NJ) offers several lines of films for radiotherapy and radiology measurements. Here we will explore Ashland's newest radiology Gafchromic film, LD‐V1 with a recommended energy range of 40−160 kVp and a recommended dose range of 20−200 mGy. In radiology applications, the focus of the work, qualities such as low‐dose sensitivity, low‐energy dependence, and high spatial precision are important. There are three lines of diagnostic Gafchromic films, which have been used for a number of tasks. The XR‐CT line, currently on its third version, excels in the computed tomography (CT) energy and dose ranges with a focus on measuring CT slice thicknesses for quality assurance testing.^12^, ^13^ The XR‐M line, also on its third version, excels under mammographic beam qualities and low energies.^14^ Finally, the XR‐RV line, also on its third version, was developed for low‐dose radiotherapy uses at low x‐ray beam energies, encountered in superficial radiotherapy.^15^, ^16^ The newest line of films, the LD line, focuses on low‐dose accuracy and quality assurance testing on radiology machines, such as planar x‐ray machines, dental equipment, or security x‐ray machines.^17^ The low‐dose focus of LD‐V1 sparked interest in its potential use for measurements of imaging doses in micro‐CT and photon‐counting computed tomography (PCCT) where the dose is lower than in a conventional CT scan.^18^, ^19^

The characterization of LD‐V1's properties under normal measurement conditions is crucial in understanding the caution required while using the first edition of this film. Despite having similar radiological properties to water, composition‐based factors could lead to discrepancies in dose output measurements. Factors in the irradiation process such as energy dependence, manufacturing batch number, and inhomogeneities in the active layer should be considered.^1^, ^13^, ^20^, ^21^ Additionally, we should consider factors in the development and readout processes such as postexposure changes, scanning orientation, temperature, ambient light sensitivity, and lateral response artifacts, among several others.^1^, ^22^, ^23^, ^24^, ^25^, ^26^ These factors could affect the analysis of dosimetric films if they are not properly accounted for. For the LD‐V1 low‐dose and low‐energy film, we focused on measuring high‐impact characteristics, namely postexposure changes, scanning orientation dependence, and energy dependence, then we tested its performance in an imaging application. Studies have already explored the sensitivity by comparing LD‐V1 to another film^27^ and the energy dependence of LD‐V1.^28^, ^29^ The energy dependence was studied in the mammographic energy range as well as across the entire manufacturer‐recommended energy range. Given the photoelectric dominance of the kilovoltage energy range, further exploration of the energy dependence in the CT/PCCT energy range (90–120 kVp) would be beneficial while also exploring other high‐impact characteristics of this film to determine its suitability.

## 2 MATERIALS AND METHODS

### 2.1 Irradiation setup

#### 2.1.1 Film calibration

**FIGURE 1:**
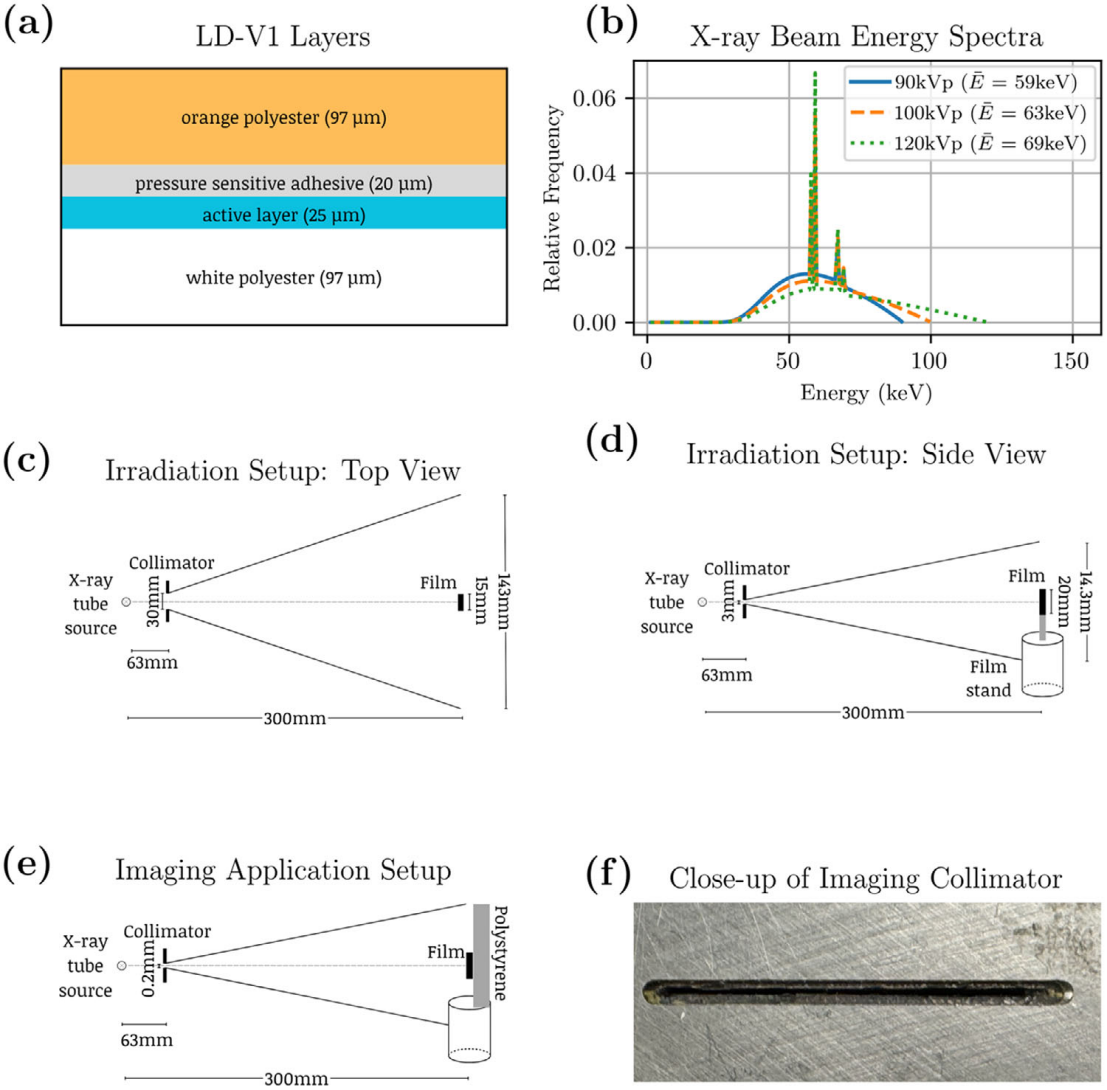
(a) Composition and thickness of each layer in LD‐V1. (b) X‐ray beam spectra used to irradiate film samples, generated with SpekPy.^30^ Filtration of 3 mm of Al and 2 mm of Ti was used for all three energies. Schematics of the film irradiation setup shown in (c) top and (d) side view. Rectangular films of 15 × 20 mm^2^ in size were laser cut from the original sheet with a notch placed in the upper left corner to track the original orientation. Film samples were suspended in air using a 3D‐printed PLA frame with minimal film obstruction. (e) Side view of imaging application setup where a 20 × 200 mm^2^ piece of film was taped to a piece of polystyrene and irradiated with a thinslit collimator (0.2 × 30 mm^2^) as shown in the close‐up in (f).

LD‐V1 is composed of four layers: an orange polyester substrate layer, followed by a thin pressure‐sensitive adhesive layer, then the active layer where the radio‐chemical reactions occur, and another polyester substrate layer dyed white (Figure 1a). The nature of this type of configuration requires reflective scanning rather than transmission scanning due to the opaque white polyester substrate layer.

A sheet of Gafchromic LD‐V1 film (LOT# 04132201) was cut into 15 × 20 mm^2^ rectangles using a laser cutter. A notch was made in the upper left corner to maintain and track the original film orientation. A custom film holder was designed and 3D printed in polylactic acid (PLA) to suspend the films in the air to achieve minimal x‐ray beam scatter. The film holder was designed to have a square opening for the film exposures, and the film samples were cut asymmetrically to allow for easy removal from the top slit of the film holder. Multiple film samples (three for doses <80 mGy and two for doses ≥80 mGy) were irradiated for each dose for each experiment to allow for averaging and an appropriate uncertainty analysis.

A MXR160/22 x‐ray tube (Comet, Flamatt, Switzerland) was used to irradiate film samples for eight different doses between 5 and 320 mGy at 90, 100, and 120 kVp with 3 mm of aluminum and 2 mm of titanium as filtration throughout the experiment. The energy spectra of the 90, 100, and 120 kVp beams with a half‐value layer (HVL) of 7.9, 8.5, and 9.5 mm Al, respectively, are shown in Figure 1b. A 3 × 30 mm^2^ lead collimator, positioned 63 mm from the source, was used to create a field size that was sufficiently large enough to cover the film sample (field size of 14.3 × 143 mm^2^ at 300 mm from the source). Doses outside the recommended range were chosen to test the dosimetric limitations of the film. Energies within the recommended range were chosen.

Exposure settings (mAs) for the desired doses were determined using in‐air measurements with a Farmer ionization chamber (PTW TN30010‐1) and an electrometer (PTW UNIDOS T10005), following the American Association of Physicists in Medicine (AAPM) TG‐61 protocol.^31^ Both the ionization chamber and electrometer were calibrated with a secondary standard ionization chamber and electrometer calibrated by the National Research Council Canada. Dose rates for the 90, 100, and 120 kVp beams ranged between 0.15 and 13.15 mGy/s, using tube currents between 0.3 and 30 mA. Film exposure times were kept as close to 30s as possible to reduce the influence of the x‐ray tube end effect, and the desired doses were delivered by varying the tube current. This procedure was repeated for all doses at all three energies. Experimental setup and distances are showcased in Figure 1c,d.

#### 2.1.2 Imaging fan beam dose measurements

In order to test LD‐V1 film for an imaging application, dose was measured using a thin‐slit lead collimator (0.2 × 30 mm^2^ placed at 63 mm in front of the source) used in a prototype table‐top photon‐counting CT imaging system composed of the same MXR160/22 x‐ray tube and a CZT detector (Redlen Technologies, Victoria, BC), for which beam filtration can manually be added using strips of the desired metals.^32^ Two‐dimensional beam profiles for the fan beam were obtained on a 20 × 200 mm^2^ piece of film from the same batch, with the original orientation tracked for scanning purposes. The film was then carefully taped to a piece of polystyrene for minimal beam obstruction and minimal backscatter. Using the same source to surface distance of 300 mm and a thin fan beam collimator (0.2 × 30 mm^2^ aperture placed at 63 mm in front of the source, resulting in a 0.95 × 143 mm^2^ field size at the film location) the film was irradiated with a 120 kVp beam with the same filtration of 3 mm Al and 2 mm Ti as used for calibration.

### 2.2 Film readout

All samples were scanned using a flatbed scanner (Epson Expression 10000XL, Suwa, Japan) scanner in reflective mode with 48‐bit color and at 150dpi for a pixel size of 169 × 169 µm^2^. The scanner was warmed up before all scanning sessions by performing five flood scans. Portrait orientation was chosen as the default orientation for consistency across all samples. Films were placed in the exact same position using blank sheets of paper taped to the scanner as a positional guide to avoid any film scanning position errors. The same 10 × 10 mm^2^ region of interest (ROI) was used for all scans regardless of scanning orientation, irradiation energy, or color channel. The ROI was repositioned for the landscape scans to match the displacement from rotating the film. Film samples irradiated with 120 kVp were scanned 1, 1.5, 2, 3, 12, 24, 48, 72, and 168 h postexposure to evaluate postexposure changes. At the 24 h time point postirradiation, the 90 and 100 kVp samples were also scanned and the 120 kVp samples were additionally rotated and then scanned in landscape orientation to determine film orientation dependence.

Scanned films were converted to dose by creating a calibration curve from the ionization chamber measurements and the net optical density (netOD) for each dose level for all three energies according to the following:

(1) $$netOD=\log_{10}\left(\frac{I_{\mathrm{flood}}}{I}\right)\;\;-\;\log_{10}\left(\frac{I_{\mathrm{flood}}}{I_{0}}\right),$$

where (*I*_0_) and (*I*) are pixel values of unirradiated and irradiated film, respectively and (*I*_flood_) is the pixel value of the flood field of the corresponding ROI. To remove artifacts due to dust or scratches in the film, *netOD* values that were not within three standard deviations of the mean were excluded (<0.3% of the data). Uncertainties in *netOD* were estimated using the standard deviation across all samples per dose after the removal of artifacts caused by dust or scratches. *NetOD* (Equation 1) was chosen rather than pixel intensity to focus on the change in optical density between the unirradiated film intensity (*I*_0_) and the irradiated film intensity (*I*). Intensities were calculated using the average intensity value for each color channel within a 10 × 10 mm^2^ ROI.

The calibration curves were fitted to the dose‐*netOD* values for each energy using a rational function (Equation 2) with coefficients *a* and *b*, calculated by a Python optimizer. *NetOD* errors were estimated from the standard deviation of the film ROI.

(2) $$D=\frac{a\cdot netOD}{b-netOD}$$

### 2.3 Film analysis

Once calibrated, films were analyzed in Python by comparing the dose‐response curves for the three color channels, portrait versus landscape orientations, and the three beam qualities. Additionally, the postexposure changes were analyzed by fitting logarithmic fits for the *netOD* changes over time for selected doses, using a Python optimizer. The linear fits were then used to calculate OD change as a function of time with respect to the calibration scan timepoint. The scan time window was defined as the time interval during which the dose will be measured to within 0.5%. The time scan window was calculated rather than measured to achieve higher precision, to avoid unnecessary light exposure to the films by scanning more frequently, and due to the high correlation achieved by the linear fits. Actual scan times were accurate to the desired scanning time within ±0.15 h for shorter scan times (≤12 h) and ±0.5 h for longer scan times (>12 h). Timestamps, accurate to the minute, were noted for every irradiation and scanned to allow for better accuracy in the analysis. Finally, the imaging application data were analyzed by plotting dose line profiles within a central ROI (roughly 20 × 10 mm^2^) in both the horizontal and vertical directions of the collimator slit.

## 3 RESULTS

**FIGURE 2:**
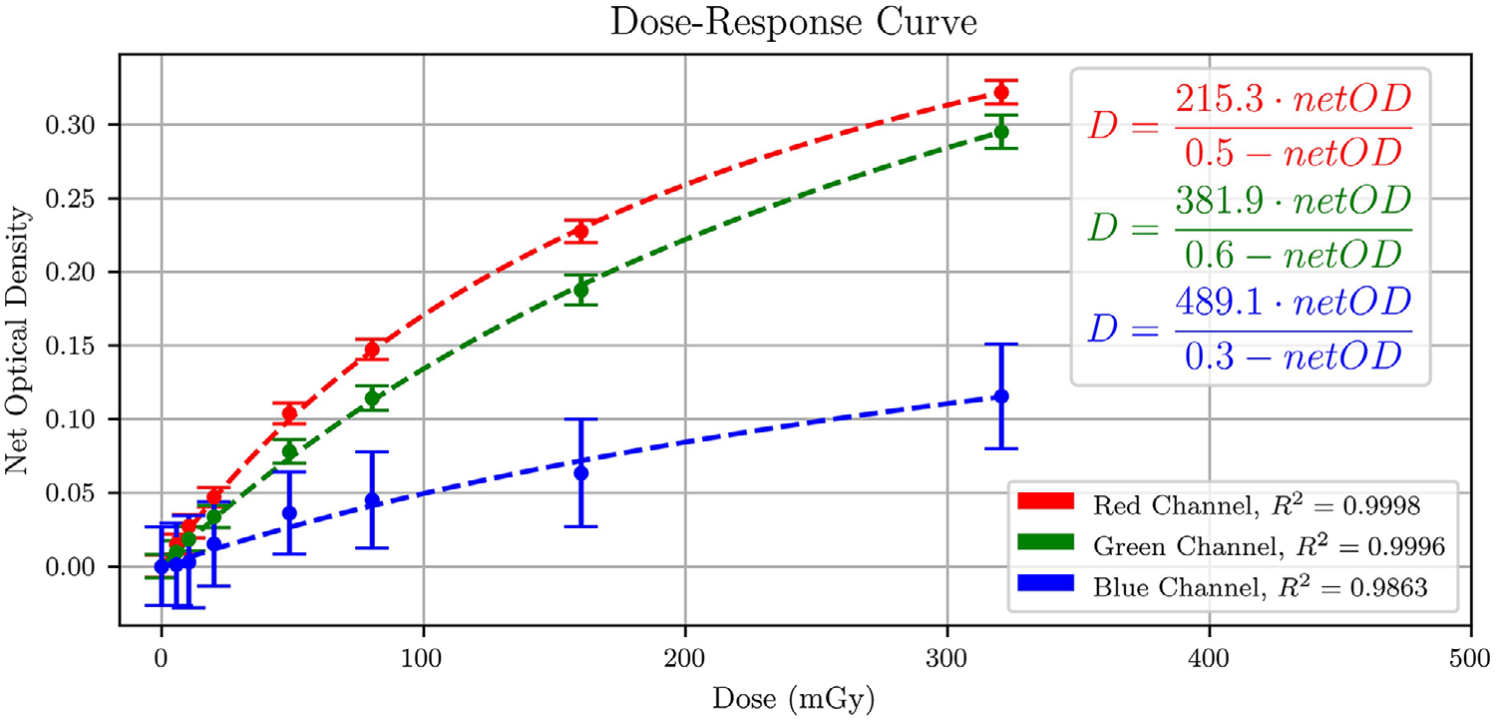
LD‐V1 dose‐response curves for the red, green, and blue channels for a 120 kVp beam. The measured net optical density was fitted using a rational function for all three channels. Uncertainties in *netOD* were estimated using the standard deviation across all samples.

The dose response of LD‐V1 films as a function of *netOD* for all three‐color channels for the 120 kVp beam alongside the rational fits is shown in Figure 2. All three fits yielded *R* ^2^ values that were larger than 0.98. The red channel had the lowest *netOD* uncertainty of 3.4% for the 160 mGy dose point compared to 5.4% and 57.6% for the green and blue channels, respectively. The red channels also covered a larger range of *netOD* within the studied dose range.

**FIGURE 3:**
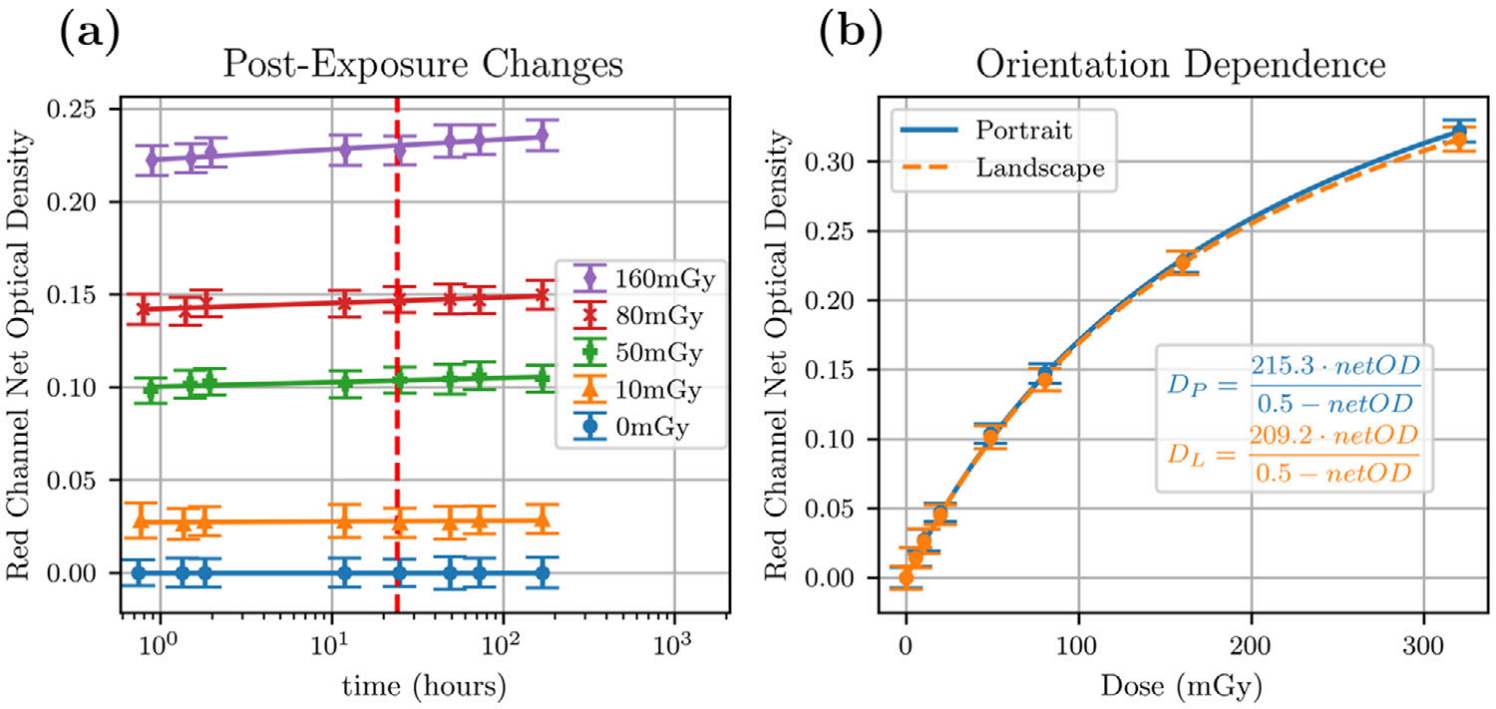
(a) Postexposure changes in the red channel *netOD* measured as a function of *log*_10_(time) for a 120 kVp beam. The vertical dashed red line indicates 24 h postexposure, the recommended film read out time point. *NetOD* errors were estimated by the standard deviation of the film region of interest. Time errors were excluded as they were precise to the minute. Linear fits were applied for each dose to demonstrate the linear progression as a function of *log*_10_(time). (b) Film response dependence as a function of scanning orientation for the red channel for the 120 kVp beam scanned 24 h postexposure. Uncertainties in *netOD* were estimated using the standard deviation across all samples.

Next, the change in *netOD* as a function of postexposure time was measured at 1, 1.5, 2, 3, 12, 24, 48, 72, and 168 h postexposure for the 120 kVp film samples (Figure 3a). The *netOD* changed logarithmically with time, leading to a 6% dose error when scanning an 80 mGy‐exposed sample at 1 h versus at 168 h, when using the 24 h dose‐response curve as reference. The dose error changed linearly with dose, with a higher dose error of 9% for 160 mGy and a lower 4% dose error for 10 mGy. Linear fits were applied to map out the changes as a function of *log*_10_ of time. Using the linear fits and an acceptable dose error of <0.5%, scanning time windows were calculated for the investigated calibration scan times (1, 1.5, 2, 12, 24, 48, 72, and 168 h). The largest scanning time windows while maintaining a dose error of <0.5% are presented in Table 1 for each of the investigated calibration scan times, with the general trend where the scanning window is 1/3 the calibration scan time. Similarly, at the 24 h scan, the orientation dependence was measured to compare the dose‐response curve in portrait versus landscape scanning orientations (Figure 3b). An average *netOD* percent difference of 2.6% was calculated, translating to an average dose error of 3.6%. The orientation dose error showed no particular dose dependence.

**TABLE 1:** Scanning time windows for varied calibration scan times, for an 80 mGy dose point.

Calibration scan time (h)	Time window (±hours) for a <0.5% dose error
1.0	0.3
1.5	0.5
2.0	0.7
12	4
24	8
48	16
72	24
168	55

*Note*: Scanning time windows were calculated using the modeled linear fit for postexposure changes of an 80 mGy dose point. Using the respective calibration scan time dose‐response curves and a dose error of <0.5%, the maximum time error (scan time window) was calculated.

**FIGURE 4:**
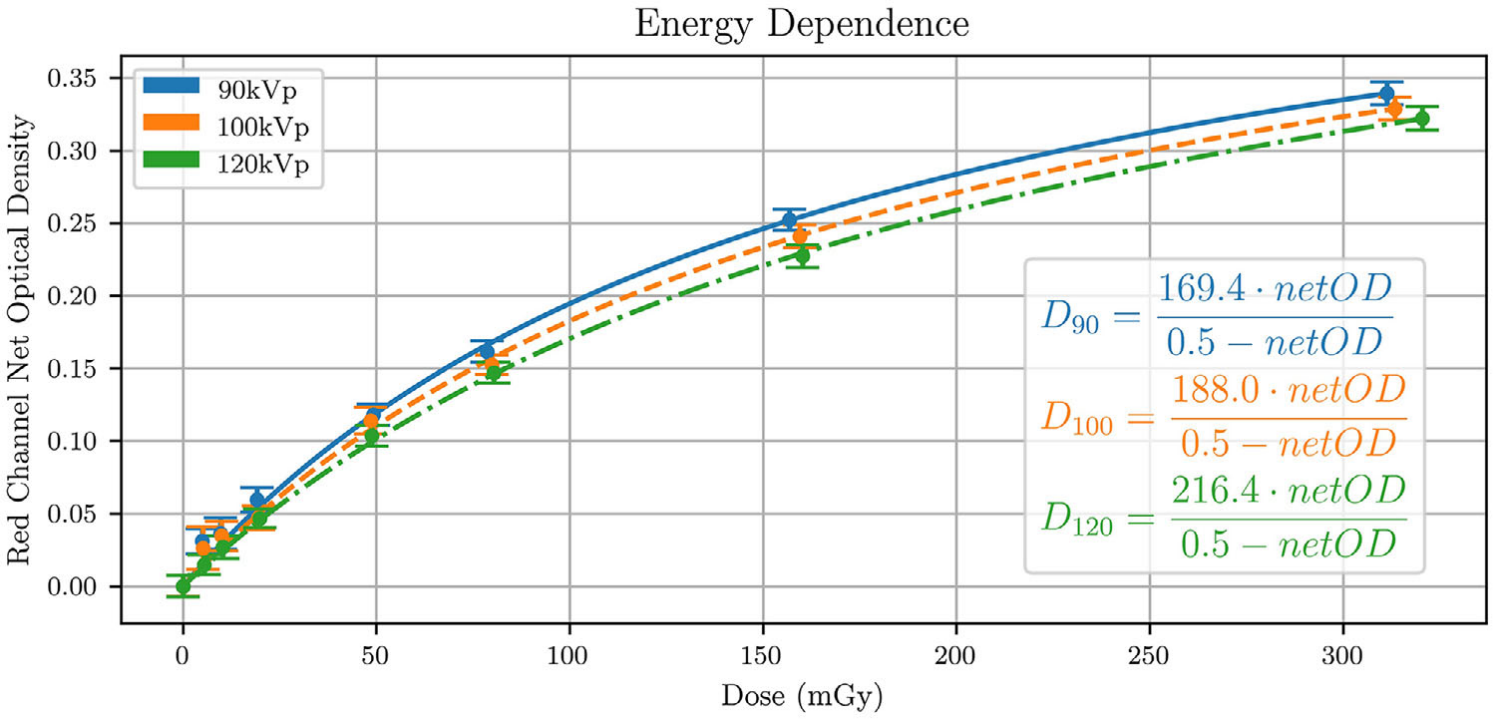
Energy dependence using three different tube voltages of 90, 100, and 120 kVp for the recommended energy range of 40–160 kVp. Markers indicate the measurements and the curves represent the rational fits. Uncertainties in *netOD* were estimated using the standard deviation across all samples.

The energy dependence of the LD‐V1 film for the 90, 100, and 120 kVp beams is plotted in Figure 4. The lower the beam energy, the higher the *netOD* for a given delivered dose. For example, for a *netOD* of 0.20, doses of 104.6, 115.4, and 127.7 mGy would be measured for 90, 100, and 120 kVp beams, respectively.

**FIGURE 5:**
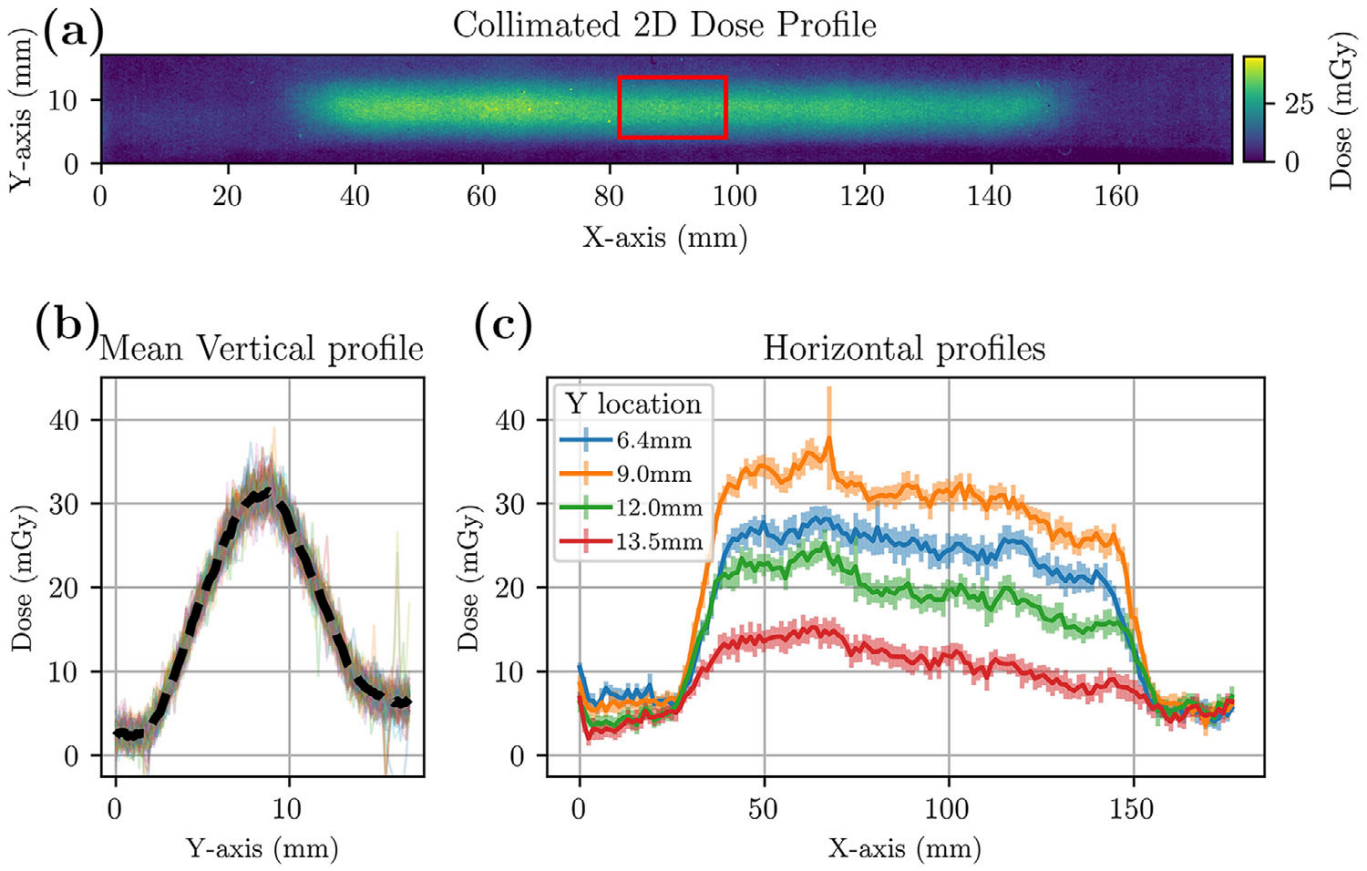
(a) 2D dose profiles for a 0.2 × 30 mm^2^ collimator aperture. The film strip was irradiated by a 120 kVp beam (HVL: 9.5 mm Al) and scanned 24 h postirradiation with 150dpi in the red channel. (b) Vertical profiles sampled from the red rectangle in (a) then averaged and displayed with a black dashed profile. (c) Horizontal samples, average profile (line) averaged over three pixels height (0.5 mm) with standard error (semi‐opaque ribbon), taken along the horizontal length of the film in (a).

Finally, LD‐V1 was tested for an x‐ray imaging application. The 30 mGy 2D dose distributions as well as dose profiles along both the vertical (Y) and horizontal (X) axes acquired with a fan beam created by an imaging collimator are shown in (Figure 5). The 2D dose distribution showed dose heterogeneity along the x‐axis and a large beam penumbra in both directions. The vertical profiles (Figure 5b) had a notable amount of noise with a dose standard deviation of 1.6 mGy and a relative noise percentage of 6%, yet were consistent in yielding a smooth average dose profile. The horizontal profiles (Figure 5c) were also noisy and demonstrated a pattern and a variation in dose along the vertical axis consistent with the profiles shown in Figure 5b.

## 4 DISCUSSION

The red channel outperformed the green and blue channels in terms of precision, sensitivity, and *netOD* range across the entire studied dose range, making it the most suitable scanning channel. The rational fit had the highest *R* ^2^ value for the red channel (*R* ^2 ^= 0.9998) and the lowest for the blue channel (*R* ^2 ^= 0.9863). The manufacturer of the LD‐V1 recommends an upper dose limit of 200 mGy to prevent film response saturation. The 200 mGy dose point was not included, despite being the manufacturer‐recommended upper dose limit. A 0.6% dose difference was calculated for the 200 mGy dose point using the full dose‐response curve presented, and a dose‐response curve was created using only the points lower than 200 mGy. Therefore, the addition of a 200 mGy dose point would likely not significantly affect the dose‐response curve measured or the *R* ^2^ value obtained. The saturation limit was tested by irradiating the film to a dose of 320 mGy. The dose‐response curves shown in Figure 2 do not show full saturation yet, suggesting that the upper dose limit could be further increased. The overall sensitivity of the film proved to be high, with a high correlation for a rational fit in the red channel. The sensitivity of LD‐V1 has been evaluated previously by Gotanda et al. by comparing it with XR‐QA2, a film known for its low‐dose sensitivity and they found, similarly, that LD‐V1 achieved high sensitivity in the range of 0 to 87.12 mGy.^27^

It was found that the LD‐V1 film is relatively stable in its development over time. A latent period of 12 h or longer is still suggested to reduce the magnitude of errors caused by the logarithmic nature of these changes. Comparatively, EBT2/3 have similar response stability times and similar logarithmic behavior of postexposure changes.^25^ Similarly, as shown in Table 1, with a latent period of 12 h or longer, the scanning time window required for doses errors <0.5% becomes significantly larger (± 4 h for 12 h) reducing the errors that may arise from time delays, such as scanner malfunction or warm up. The linear fits in Figure 3a demonstrated that the postexposure changes were more prominent at higher doses. To keep dose errors below 0.5%, it was determined that the exposure‐to‐scan time should be within 24 ± 8 h for a 24 h scan time or 2.0 ± 0.7 h for a 2 h scan time, as shown in Table 1. Given that the width of the time window scales up with a longer exposure‐to‐scan time, a 24 h scan is the most convenient for its large scan time window and for scheduling purposes in that the user does not need to wait several days and middle‐of‐the‐night scans can be easily avoided. LD‐V1 offers more flexibility compared to the EBT models, which typically allow a 24 ± 2 h window to scan the films for a similar dose error (0.6%).^26^ Additionally, LD‐V1 offers a scan time window that scales linearly with time, while EBT films the scanning window shortens for longer exposure‐to‐scan times.^26^

The scanning orientation caused a change in the dose‐response curve with a mean dose error of 3.6% and no particular dose dependence. Orientation should be tracked and maintained while scanning to obtain a more precise dose measurement. In terms of film orientation dependence, LD‐V1 performs similarly compared to low‐dose films such as XR‐QA2, which have relative dose differences of roughly 2%.^33^ On the other hand, compared to high‐dose radiotherapy films, LD‐V1 outperforms EBT3 (relative dose difference of 7%) and performs similarly to EBT4 (4%).^22^

A significant energy dependence was measured for the 90, 100, and 120 kVp beams. Low‐energy beams presented a higher film response compared to high‐energy beams. The measured energy dependence follows the behavior of other Gafchromic films, such as XR‐QA2 and XR‐RV3.^33^, ^34^, ^35^ However, Nakajima et al. tested the energy dependence of LD‐V1 for mammography beam qualities and noticed the opposite effect from what we observed; higher‐HVL beams resulted in an increased dose response compared to lower‐HVL beams.^29^ The beam qualities Nakajima et al. used were much softer than the ones we used with HVLs ranging from 0.32 to 0.61 mm of Al, while ours ranged from 7.9 to 9.5 mm of Al, suggesting that the energy dependence is not consistent across the recommended energy range (40–160 kVp). It should be noted that for EBT3 and EBT4 films, lower‐energy 70 kVp x‐ray beams (filtered with 1.26 mm Al) resulted in lower dose response compared to higher‐energy 6MV beams.^20^ Mbewe also explored the energy dependence of LD‐V1 and found a significant energy dependence (>10% error) for doses <50 mGy and a dose error of 8% for doses >50 mGy for beam qualities as low as 60 kV (HVL of 1.32 mm Al) and as high as 180 kV (HVL of 0.54 mm Cu).^28^ The high dose error likely resulted from a large dose uncertainty (8%) and the wide range in beam qualities. Given the conflicting results, the energy dependence of this film should be explored further. As a result, the beam quality used for calibration should match the beam quality used for measurements to avoid significant dose errors. For example, assuming a 90 kVp calibration curve for a 120 kVp, 80 mGy measurements, the difference in dose could be as high as 17 mGy, resulting in a 21% dose measurement error.

The LD‐V1 film was used for a diagnostic imaging application to measure the dose for a fan beam used for a table‐top photon‐counting CT imaging system. The spatial resolution of the LD‐V1 film scanned with a pixel size of 169 µm was high, however, the dose map with values of 20−30 mGy had a relative noise of 6%, which was highlighted in the profiles shown in Figure 5, suggesting that there is a notable trade‐off between spatial resolution and noise for this film.

The mean vertical profile in Figure 5b was smooth, however, it was asymmetric. Lower doses were observed at lower *Y*‐values compared to the higher *Y*‐values. This behavior was consistent across the entire 2D dose profile and is likely the result of the imperfect collimator manufacturing. Individual profiles contained spikes due to the high scanning resolution used as this impacted the background film to a similar extent and could easily be smoothed at the expense of spatial resolution.

The noise and variation along the horizontal profiles in Figure 5c followed a general pattern that can be seen for all Y locations. This variation can be explained by the geometry of the collimator which features an uneven surface within the collimator slot. A consistent pattern in the profile was observed using a photon counting detector, proving that the dose pattern was related to the geometry of the collimator and unrelated to the film spatial resolution. As a result, the spatial beam capabilities of LD‐V1 are significantly limited by the desired level of noise as well as the equipment used. Precise equipment should be used in cases where spatial resolution cannot be sacrificed, such as with SBRT measurements.

## 5 CONCLUSIONS

A number of properties of LD‐V1 Gafchromic film were evaluated, namely the dose‐response curve across all three color channels, the postexposure changes, scanning orientation dependence and energy dependence. The LD‐V1 film was tested in an x‐ray imaging application. The LD‐V1 low‐dose film provided a wide range of *netOD* values across the recommended dose range in the red channel for doses ranging from 0 to 320 mGy with no signs of saturation. Additionally, the postexposure changes revealed that the scanning time window is rather flexible at ± 8 h for an exposure‐to‐scan time of 24 h. On the shorter end, a scan time of 2 h offers less flexibility with a scanning time window of ± 0.7 h. This suggests that a longer exposure‐to‐scan time is recommended in order to increase the width of the scanning window for a reasonable dose error (<0.5%), allowing room for error in time without a significant error in dose. Scanning orientation dependence was low, with an average *netOD* difference of 2.6% between portrait and landscape orientations. LD‐V1 demonstrated a notable energy dependence with dose errors in the range of 20% for a 100 mGy measurement in the range of 90 to 120 kVp and should be calibrated separately for each beam quality.

Due to its good spatial resolution and low‐dose response, this film is useful for performing x‐ray imaging dosimetric and geometric analyses. The low‐dose response allows for faster setup validation by drastically shortening the exposure time required to achieve a reasonable contrast with a kV beam compared to other Gafchromic films, such as the EBT3 or EBT4 models.

## AUTHOR CONTRIBUTIONS

*Conception and design*: Oliva Masella and Magdalena Bazalova‐Carter. *Data collection*: Oliva Masella and Kevin J. Murphy. *Data analysis and manuscript writing*: Oliva Masella. *Edits and final approval of manuscript*: Oliva Masella, Kevin J. Murphy, and Magdalena Bazalova‐Carter.

## CONFLICT OF INTEREST STATEMENT

The authors declare no conflicts of interest.
