# Correction to “Characterization of a new low‐dose and low‐energy Gafchromic film LD‐V1”

**DOI:** 10.1002/acm2.70241

**Published:** 2025-09-01

Masella O, Murphy KJ, Bazalova‐Carter M, Characterization of a new low‐dose and low‐energy Gafchromic film LD‐V1. *J Appl Clin Med Phys*. 2024;25(12):e14531. https://doi.org/10.1002/acm2.14531

The corresponding author’s name has been updated as Olivia Masella in the author byline and the author contributions section at the end of this article. The online version of this article has been corrected accordingly.

